# Assessing Autism in Adults: An Evaluation of the Developmental, Dimensional and Diagnostic Interview—Adult Version (3Di-Adult)

**DOI:** 10.1007/s10803-017-3321-z

**Published:** 2017-11-07

**Authors:** William Mandy, Kiri Clarke, Michele McKenner, Andre Strydom, Jason Crabtree, Meng-Chuan Lai, Carrie Allison, Simon Baron-Cohen, David Skuse

**Affiliations:** 1 The Research Department of Clinical, Educational and Health Psychology, University College London, London, WC1E 6BT UK; 2 The Division of Psychiatry, UCL, London, WC1E 6BT UK; 3 Autism Research Centre, Department of Psychiatry, University of Cambridge, 18B Trumpington Road, Cambridge, CB2 8AH UK; 4 Child and Youth Mental Health Collaborative at the Centre for Addiction and Mental Health and The Hospital for Sick Children, and Department of Psychiatry, University of Toronto, Toronto, M5T 1R8 Canada; 5 Department of Psychiatry, National Taiwan University Hospital and College of Medicine, Taipei, 10051 Taiwan, Republic of China; 6 Institute of Child Health, UCL, Guilford Street, London, WC1N 1EH UK

**Keywords:** Autism spectrum conditions (ASC), Autism spectrum disorder (ASD), Diagnostic and statistical manual, fifth edition (DSM-5), Assessment and diagnosis, Adults

## Abstract

We developed a brief, informant-report interview for assessing autism spectrum conditions (ASC) in adults, called the Developmental, Dimensional and Diagnostic Interview-Adult Version (3Di-Adult); and completed a preliminary evaluation. Informant reports were collected for participants with ASC (n = 39), a non-clinical comparison group (n = 29) and a clinical comparison group (n = 20) who had non-autistic mental health conditions. Mean administration time was 38 min (50 min for ASC). Internal consistency (αs ≥ 0.93) and inter-rater agreement (ICCs ≥ 0.99) were high. When discriminating ASC from non-ASC, the 3Di-Adult showed excellent sensitivity (95%) and specificity (92%). The 3Di-Adult shows promise as a psychometrically sound and time-efficient interview for collecting standardised informant reports for DSM-5 assessments of ASC in adults, in research and clinical practice.

## Introduction

Autism spectrum condition (ASC) (also known as ‘autism spectrum disorder’^1^) is an early-onset neuro developmental syndrome that affects approximately 1% of the population, characterised by lifelong difficulties with social communication, social reciprocity, flexibility and sensory processing (American Psychiatric Association 2013). A substantial proportion of people with ASC, especially those with fluent language and normal-range IQ, are not identified in childhood, and enter adulthood without a diagnosis (Baron-Cohen et al. 2009; Lai and Baron-Cohen 2015).

Undiagnosed adults are at high risk of experiencing functional and emotional difficulties as a result of their ASC (Lai and Baron-Cohen 2015). Autistic people who were only diagnosed with ASC after they had entered adulthood consistently recall that, prior to their diagnosis, they had substantial social, sensory and flexibility difficulties that impacted negatively on their wellbeing (e.g., Lewis 2016; Portway and Johnson 2005). Such reports are corroborated by the observation that around a quarter of adults presenting to specialist services for obsessive compulsive disorder (Wikramanayake et al. 2017) and anorexia nervosa (Westwood et al. 2017) have an undiagnosed ASC.

Further, a consistent finding from studies of adult-diagnosed ASC is that, prior to diagnosis, participants’ autistic difficulties had been misunderstood and poorly supported, with negative consequences for their wellbeing and functioning (e.g., Bargiela et al. 2016; Portway and Johnson 2005). Thus, it appears that the lack of an appropriate diagnosis compounds the challenges that stem from having ASC. Conversely, those who receive a late (i.e., adult) ASC diagnosis often report that this brings diverse benefits, including improved access to appropriate services, greater self-understanding and self-acceptance, more understanding from others, and the chance to join a community of autistic adults (Bargiela et al. 2016; Lai and Baron-Cohen 2015; Portway and Johnson 2005; Powell and Acker 2016; Pushon et al. 2009). As such, there is an urgent need to provide effective assessment protocols to identify autism in adulthood, and this relies upon the existance of valid ASC diagnostic instruments for adults (Department of Health 2015). Currently such instruments are less numerous and less well validated than those designed for children and adolescents [Howlin and Moss 2012; National Institute for Health and Clinical Excellent (NICE) 2012].

Making a first diagnosis of an ASC in adulthood is challenging for a range of reasons (Lai and Baron-Cohen 2015). Diagnostic rules require that symptoms be present from early childhood, so accurate historical information is essential but often hard to attain. Even current symptoms can be difficult to assess, as these can be obscure in adults who have developed ways of camouflaging and/or compensating for their autistic characteristics (Hull et al. 2017). An additional challenge to assessment is that some adults with ASC struggle to provide self-reports of their difficulties (Bishop and Seltzer 2012). Furthermore, ASC usually presents as part of a complex clinical picture involving co-occurring mental health conditions, with depression, anxiety and attention deficit/hyperactivity disorder being the most common (Moss et al. 2015; Howlin et al. 2014). This can result in diagnostic overshadowing, whereby clinicians mistakenly attribute autistic difficulties (e.g., social difficulties) to a co-occurring mental health condition (e.g., anorexia nervosa) (Mandy and Tchanturia 2015). The converse effect can also occur, whereby the symptoms of a mental condition (e.g., rituals associated with obsessive compulsive disorder) are mistakenly interpreted as indicators of ASC.

To overcome such challenges, informant report information is essential, as part of a broader multi-perspective assessment that also incorporates self-reports and direct observation from clinicians (Pilling et al. 2012). Here we define an informant as a family member or other third party who has known the person being assessed for ASC since childhood, and can provide information about that person’s past and current autism-relevant characteristics (NICE 2012). Currently, for clinical practice and research, there is a need for an informant interview that was designed specifically for assessing adults, is relatively brief to administer (i.e., can be conducted in approximately one hour), and reflects current (i.e., DSM-5) diagnostic criteria for ASC. Therefore we developed and evaluated such an instrument: the Developmental, Diagnostic and Dimensional Interview-Adult version (3Di-Adult). This structured, informant interview has a diagnostic algorithm that yields quantitative scores for each element of the DSM-5 autism dyad, namely ‘Social Communication and Social Interaction’ and ‘Restricted, Repetitive Patterns of Behaviour, Activities or Interests’ dimension.

In the current study we sought to conduct a preliminary investigation of the 3Di-Adult’s psychometric properties, via consideration of its reliability and validity. To estimate reliability, we examined the internal consistency and the level of inter-rater agreement for the 3Di-Adult DSM-5 algorithm. Validity was tested by investigating the 3Di-Adult’s ability to distinguish individuals with ASC from those without ASC in the general population, and from those without ASC who were receiving psychiatric care for other mental health difficulties. Specifically, this is an investigation of criterion validity, with diagnostic status being the ‘gold standard’ (i.e., criterion) against which the 3Di-Adult was tested (Barker et al. 2015; Mokkink et al. 2010). In addition to investigating reliability and validity, we also sought to explore the utility of the 3Di-Adult. To this end we measured how long it took to administer; and examined patterns of missing data to learn whether its use was impeded by informants struggling to provide relevant information.

## Methods

### Participants

Three groups were recruited: (1) participants with ASC (n = 39); (2) people without ASC from the general population (‘non-clinical comparison group’) (n = 29); and (3) people without ASC who were receiving psychiatric care from the UK National Health Service (NHS) for mental health difficulties (‘clinical comparison group’) (n = 20). This sample size (N = 88) is in line with recommendations (N = 50–100) from the Consensus-based Standards for selection of health status Measurement Instruments (COSMIN) guidelines for studies using Classical Test Theory (CTT) (Mokkink et al. 2010). Sample characteristics are displayed in Table 1. The groups did not differ in terms of age and IQ but the ASC group, which was mainly male, had a higher proportion of men than the clinical comparison group, in which females predominated.

**Table 1:** Characteristics of the sample (N = 88)

	Autism spectrum condition (ASC) (n = 39)	Non-clinical comparison (NCC) (n = 29)	Clinical Comparison (CC) (n = 20)	Group differences
Proportion male	77%	59%	30%	p = .002 ASC > CC, NCC > CC
Age in years				
Mean (SD)	32.9 (12.0)	31.2 (9.9)	28.9 (8.8)	ns
95% CI	29.1–36.8	26.5–36.0	25.5–32.4	
Range	18–59	21–50	18–52	
Full-scale IQ^a^				
Mean (SD)	109.8 (14.4)	116.5 (10.7)	107.6 (13.0)	ns
95% CI	104.3–115.3	112.1–120.8	101.0–114.3	
Range	72–138	89–137	88–134	

^a^IQ estimate was available for 72 of the 88 participants: ASC n = 29; NCC n = 26; CC n = 17

All participants were required to be aged 18 years or over. Potential participants were excluded if they had a learning disability (as indicated by an estimated IQ under 70) or if there was no informant available to complete their 3Di-Adult. ASC participants entered the study via two routes: (1) participation in one site of the MRC Autism Imaging Multicentre Study (AIMS) (n = 12); (2) attendance of two NHS adult ASC clinics in South East England (n = 27). To be included in the ASC group, NHS participants were required to meet criteria for ‘autism spectrum’ or ‘autism’ on the Autism Diagnostic Observation Schedule (ADOS) Module 4 (Lord et al. 2000), with this diagnosis being confirmed by clinician consensus to avoid the inclusion of ADOS false positive cases. AIMS participants were diagnosed according to the protocol of that study, with all 12 being classified as having ‘autism’ on the Autism Diagnostic Interview-Revised (ADI-R; Lord et al. 1994) (Lai et al. 2014). Participants in the clinical comparison group were required to have a diagnosis of a mental disorder for which they were receiving NHS treatment at the time of the study. This group were receiving services for mixed anxiety and depression (n = 8), depression (n = 4), anxiety (n = 4), borderline personality disorder (n = 3), and a psychotic disorder (n = 1). Participants in the non-clinical comparison group were excluded if they reported any current mental health difficulties. Participants were excluded from either comparison group if any current or previous concerns had been raised about them having an ASC, unless such concerns had been ruled out by a formal multidisciplinary ASC assessment.

### Measures

#### Developmental, Diagnostic and Dimensional Interview—Adult Version (3Di-Adult)

The 3Di-Adult was developed from the childhood/adolescent version of the 3Di (Skuse et al. 2004). The original (i.e., child/adolescent) 3Di has strong psychometric properties. Its reliability is excellent, demonstrated by high levels of inter-rater and test–retest agreement (intraclass correlation coefficients > 0.86), and it possesses criterion validity in relation to both clinician diagnosis and the ADI-R (Lord et al. 1994; Skuse et al. 2004).

The 3Di-Adult was developed using the following process. First, an initial item pool was taken from the diagnostic algorithm of the original 3Di, as these items had already been empirically identified as being especially discriminating (Skuse et al. 2004; Santosh et al. 2009). Second, these items were mapped onto DSM-5 criteria, based on discussions within the study team, which incorporates clinical and research expertise in autism assessment across the lifespan. For example, the 3Di item on having a ‘rigid day to day routine’ was assigned to DSM-5 criterion B2 (‘Insistence on sameness, inflexible adherence to routines, or ritualized patterns or verbal/nonverbal behaviour’), whereas the 3Di item on being ‘distressed by everyday sounds’ was judged to represent DSM-5 criterion B4 (‘Hyper- or hypo-reactivity to sensory input or unusual interests in sensory aspects of the environment’). Third, based on discussion within the study team supported by pilot analyses of existing 3Di data, items were then sorted into those that would be most discriminating and/or appropriate in childhood, and those that would work when used to assess adult symptoms. The items deemed most appropriate for children (n = 21) were phrased as historical questions in the 3Di-Adult (e.g., questions on imitation during preschool years, being invited for play dates, lining up toys). The other 3Di algorithm items (n = 31) were judged to be applicable to adult life, and so were phrased as assessing current behaviour (e.g., questions on conversation, use of gesture, resistance to change). Fourth, 17 new questions were created based upon knowledge of the ASC phenotype in adulthood (e.g., on getting into trouble due to being ‘easily led’ and gestures appearing exaggerated or ‘put on’) and to ensure that all aspects of DSM-5 criteria were covered (e.g., additional questions about sensory reactivity). The resultant 3Di-Adult comprises 69 questions in total, 48 of which assess current behaviour, and 21 of which are historical, covering the assessee’s behavioural characteristics between birth and adolescence.

**Fig. 1:**
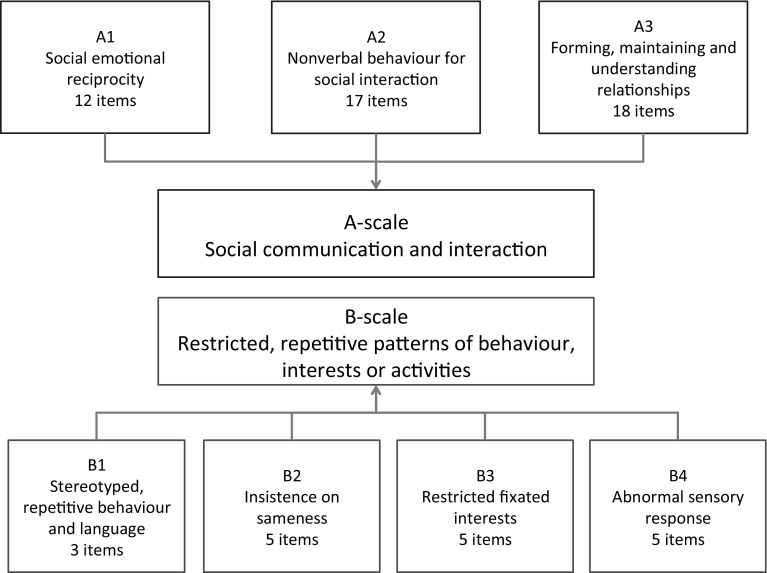
Structure of the 3Di-Adult’s DSM-5 diagnostic algorithm

The 3Di-Adult is a structured interview, as its 69 questions are asked verbatim, in a set order. Nevertheless, its administration is a collaborative dialogue between the interviewer and interviewee that serves to clarify the meaning of questions asked, and of answers provided. Reflecting the fact that the 3Di-Adult is highly structured, it is intended to be suitable for administration both in person and by telephone. This acknowledges the fact that in the assessment of adults, parental report can sometimes be attained over the telephone but not in a face-to-face interview (Ward-King et al. 2010). Of the 69 3Di-Adult interview questions, 65 are included in a DSM-5 diagnostic algorithm. The remaining four questions measure early developmental milestones and play. Questions included in the algorithm are arranged into two main scales, the ‘A-scale’ which measures the DSM-5 ‘Social Communication and Social Interaction’ dimension, and the ‘B-scale’ which reflects the DSM-5 ‘Restricted, Repetitive Patterns of Behaviour, Activities or Interests’ dimension. The A-scale and B-scale are comprised of separate subscales reflecting the DSM-5 diagnostic criteria, forming a total of seven subscales (i.e., three for the A-scale and four for the B-scale). The arrangement of questions within the subscales, and how the subscales load onto the A- and B-scales is displayed in Fig. 1.

Questions are scored on either a three point (0 = Often, 1 = Sometimes, 2 = Never) or four point Likert scale (0 = No, 1 = Yes, minimal, 2 = Yes, persistent, 3 = Yes, persistent with functional impairment). All questions receiving a score of 3 are recoded to 2 when calculating algorithm scores in order to ensure that all items within a subscale carry equivalent weight. Scores for each of the seven subscales are generated by averaging the responses to each of the relevant questions, so that each subscale has a range from 0 to 2. Overall scores for the A-scale and the B-scale are generated by summing subscale scores. Thus, the A-scale, which is the sum of three subscales, has a maximum score of six, and the B-scale, which draws on four subscales, has a maximum score of eight. On the 3Di-Adult DSM-5 algorithm, higher scores indicate a greater level of autistic symptomatology.

#### Wechsler Abbreviated Scales of Intelligence (WASI)

The four-subtest WASI was used to estimate full-scale IQ for all AIMS participants. This is a well-validated measure that has been used extensively to assess the IQ of adults with and without ASC (Wechsler 1999).

#### Test of Premorbid Functioning—UK Version (TOPF)

The TOPF was used to estimate the full-scale IQ of non-AIMS participants for whom a clinically ascertained IQ estimate was not available. This is a brief measure for individuals aged 16–89 years (Wechsler 2009). It has been shown to demonstrate good internal reliability (0.95), good test–retest reliability (0.89–0.95), and high correlation (0.81) with full-scale IQ score as measured by the Wechsler Adult Intelligence Scale- fourth edition (WAIS-IV; Wechsler 2008). It has been validated for use in various clinical populations including individuals with ASC and major depressive disorder (Wechsler 2009).

### Procedure

Of the ASC participants recruited from the NHS, 15 cases had completed their assessment within the past two years and consented for their data (including 3Di-Adult scores) to be included in research (referred to as ‘past attenders’). A further 12 cases were recruited from the NHS at the time that they attended the clinic for assessment (‘current attenders’). AIMS participants were contacted by post and invited to join the study. The clinical comparison group was recruited from three sources in the NHS: a primary care (first-line) adult mental health service, an early intervention for psychosis service, and from cases who attended one of the NHS ASC assessment services and were judged to have at least one mental disorder but not to have ASC. The non-clinical comparison group was recruited via adverts placed around a London university campus and, in two cases, from the control group of AIMS. The study was approved by an NHS Ethics Committee (14/LO/1134), by the Cambridge University Ethics Committee, and by relevant local Research and Development departments.

The 3Di-Adult was carried out with an informant for all participants. Informants were mothers in the majority of cases (92% of NHS current attenders/AIMS ASC group, 92% of non-clinical comparison group, and 70% of clinical comparison group). In the remaining instances fathers or sisters completed the 3Di-Adult. Interviews were usually carried out over the telephone (80% of NHS current attenders/AIMS ASC group, 85% of non-clinical comparison group, and 85% of clinical comparison group). For 22 of the 24 (92%) NHS current attenders/AIMS ASC interviews, researchers conducted the 3Di-Adult, with a specialist clinician administering the remaining two interviews. All 3Di-Adult interviews for NHS past attender ASC cases were conducted face-to-face by clinicians, who were either psychiatrists or graduate level psychologists. Researchers and clinicians conducting the 3Di-Adults included in the study had all been trained in its use. Half of the interviews in this study (n = 44/88) were audio recorded (ASC n = 10, non-clinical comparison n = 15, clinical comparison n = 19) in order to assess inter-rater reliability. All recordings were rescored by a psychology undergraduate trained in using the 3Di-Adult, who was blind to the participant group.

To assess full-scale IQ, all AIMS participants completed the WASI. For eight participants with ASC recruited from the NHS, IQ scores were available from their clinical assessments, from the WASI (n = 2)^16^ or the Wechsler Adult Intelligence Scales, fourth edition (n = 6)^18^. All other participants were asked to complete a TOPF to estimate their IQ.

### Analysis

Analyses were conducted in SPSS, version 22. Group differences were tested using ANOVA, bootstrapped in 1000 samples to handle the non-normality of some variables. Group differences on the 3Di-Adult are reported as Cohen’s d to provide a standardised description of the observed effects. By convention, Cohen’s d = 0.2 is considered small, Cohen’s d = 0.5 is medium and Cohen’s d = 0.7 is a large effect (Cohen 1992). Correlations were calculated using Spearman’s Rho (r_s_) to protect against biases due to non-normal distributions and outliers. Inter-rater reliability was assessed using two-way random, single measures intra-class correlation coefficients, and Cronbach’s alpha was used to index internal consistency. Receiver Operating Characteristics (ROC) curves were generated to examine the ability of the 3Di-Adult to discriminate between the ASC and the comparison groups, and to set optimal thresholds for ASC caseness, designed to maximise sensitivity and specificity.

## Results

### Administration Time and Missing Data

Within the ASC group, mean administration time was 50 min (range 23–75 min). The interview was quicker to compete for the clinical (mean 30, range 15–58 min) and non-clinical (mean 26 min, range 16–36 min) comparison groups. For algorithm items, the median number of missing responses per item was two out of 88 (range 0–10). When calculating subscale scores we prorated when data for at least half of contributing items were available. In the sample of 88, there was only one participant (in the clinical comparison group) for whom a subscale score (B1: repetitive motor movements or speech) could not be calculated due to missing data.

### Reliability of the 3Di-Adult DSM-5 Algorithm

Convention suggests that reliability coefficients over 0.9 represent excellent reliability (Barker et al. 2015). Internal consistency was high for the A-scale (α = 0.97) and B-Scale (α = 0.93). Similarly, inter-rater reliability of the A-scale (r = .99) and B-scale (r = .99) was very high.

### Criterion Validity of the 3Di-Adult DSM-5 Algorithm

#### Comparison of DSM-5 Algorithm Scores

**Fig. 2:**
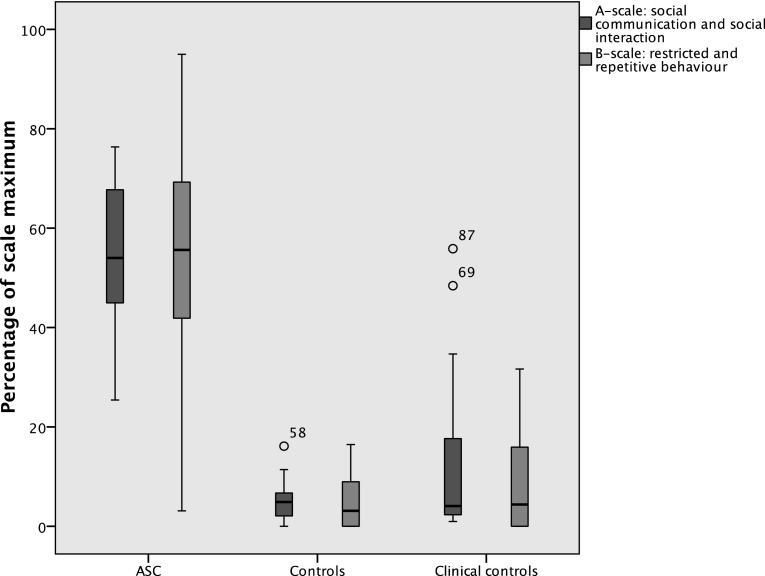
Scores on the 3Di-Adult’s DSM-5 algorithm by group

Mean scores for the 3Di-Adult A-scale and B-scale and for their constituent subscales, for each of the groups, are displayed in Table 2. There were moderate to large associations between A- and B-scale scores in both the ASC group (r_s_ = 0.37, p = .021) and amongst those without ASC (r_s_ = 0.46, p = .001). The ASC group was found to score significantly higher than both of the comparison groups for all subscales, with large effect sizes in all cases. The clinical and non-clinical comparison groups did not differ on the A-scale or B-scale or any of the subscales. Figure 2 shows the distribution of scores on the 3Di-Adult A- and B-scales for each group.

**Table 2:** Scores on the 3Di-Adult DSM-5 algorithm by group

	Autism spectrum condition (n = 39)	Non-clinical comparison (n = 29)	Clinical Comparison (n = 20)	Significance	Group difference expressed as standardised effect size (Cohen’s d)		
					*ASC v NCC*	*ASC v CC*	*NCC v CC*
*A-scale*							
Social communication and social interaction							
Mean (SD)	3.3 (0.8)	0.3 (0.2)	0.7 (1.0)	p < .001	5.1*	2.9*	− 0.6
95% confidence interval	3.0–3.6	0.2–0.4	0.3–1.2				
Range (0–6)	1.5–4.6	0.0–1.0	0.1–3.4				
A1: social emotional reciprocity							
Mean (SD)	1.1 (0.4)	0.1 (0.1)	0.3 (0.4)	p < .001	3.4*	2.0*	− 0.7
95% confidence interval	1.0–1.2	0.1–0.2	0.1–0.4				
Range (0–2)	0.2–1.9	0.0–0.6	0.0–1.3				
A2: deficits in nonverbal behaviour used for social interaction							
Mean (SD)	1.0 (0.4)	0.1 (0.1)	0.2 (0.3)	p < .001	3.1*	2.6*	− 0.4
95% confidence interval	0.9–1.1	0.0–0.1	0.4–0.6				
Range (0–2)	0.3–1.8	0.0–0.3	0.0–1.8				
A3: deficits in forming, maintaining and understanding relationships							
Mean (SD)	1.2 (0.3)	0.1 (0.1)	0.3 (0.4)	p < .001	4.9*	2.5*	− 0.7
95% confidence interval	1.1–1.3	0.1–0.2	0.1–0.5				
Range (0–2)	0.7–1.8	0.0–0.4	0.0–1.3				
*B-scale*							
Restricted repetitive patterns of behaviour, activities or interests							
Mean (SD)	4.3 (1.8)	0.4 (0.4)	0.7 (0.9)	p < .001	3.0*	2.5*	− 0.4
95% confidence interval	3.8–4.9	0.2–0.5	0.4–1.1				
Range (0–8)	0.2–7.6	0.0–1.3	0.0–2.5				
B1: stereotyped or repetitive movements^a^							
Mean (SD)	1.0 (0.5)	0.1 (0.2)	0.1 (0.2)	p < .001	2.4*	2.4*	0.0
95% confidence interval	0.9–1.2	0.0–0.2	0.0–0.2				
Range (0–2)	0.0–2.0	0.0–0.5	0.0–0.8				
B2: insistence on sameness							
Mean (SD)	1.3 (0.7)	0.0 (0.1)	0.3 (0.4)	p < .001	2.6*	1.75*	-1.0
95% confidence interval	1.1–1.5	0.0–0.1	0.1–0.5				
Range (0–2)	0.00–2.00	0.0–0.7	0.0–1.3				
B3: restricted fixated interests							
Mean (SD)	1.2 (0.6)	0.2 (0.2)	0.2 (0.3)	p < .001	2.2*	2.1*	0.0
95% confidence interval	1.0–1.4	0.1–0.3	0.1–0.3				
Range (0–2)	0.0–2.0	0.0–0.8	0.0–1.2				
B4: abnormal sensory response							
Mean (SD)	0.8 (0.6)	0.0 (0.1)	0.2 (0.2)	p < .001	1.9*	1.3*	-1.3
95% confidence interval	0.6–1.0	0.0–0.1	0.1–0.3				
Range (0–2)	0.0–2.0	0.0–0.4	0.0–0.6				

^a^NCC group n = 26, due to one case lacking sufficient data to calculate B1 score, *p < .001

ROC curves were generated to analyse the ability of the 3Di-Adult A- and B-scales to discriminate between: (1) ASC and all comparison participants; (2) ASC and clinical comparison participants; and (3) ASC and non-clinical comparison participants. The 3Di-Adult showed high AUCs when discriminating ASC from all comparison participants [A-scale AUC = 0.98, 95% CI (0.96, 1); B-scale AUC = 0.97, 95% CI (0.93, 1)]. This reflected high accuracy when discriminating people with ASC from clinical comparison participants [A-scale AUC = 0.95, 95% CI (0.89, 1); B-scale AUC = 0.96, 95% CI (0.91, 1)] and from non-clinical comparison participants [A-scale AUC = 1, 95% CI (1, 1); B-scale AUC = 0.98, 95% CI (0.94,1)].

#### Sensitivity and Specificity

Using data for all three groups, with the two comparison groups combined, cut points which maximised both sensitivity and specificity for both the A- and B-scale were identified. For the A-scale (range 0–6) this was a score of 1.4 and for the B-scale (range 0–8) this was a score of 1. In order to be categorised as having ASC by the 3Di-Adult a person must score above the cut-off on both scales, in line with DSM-5 criteria. The number of cases correctly categorised by the 3Di-Adult DSM-5 algorithm using these thresholds is displayed in Table 3. There were two ASC participants who were not identified by the 3Di-Adult DSM-5 algorithm. In both these false negative cases they scored above threshold on the A-scale, but not the B-scale.

**Table 3:** Agreement between clinical and 3Di-Adult DSM-5 algorithm

	Participant group		
	ASC	Non-clinical comparison	Clinical comparison
3Di-adult diagnosis			
Non-ASC	2 (5.1%)	29 (100%)	16 (80.0%)
ASC	37 (94.9%)	0 (0%)	4 (20.0%)

*ASC* autism spectrum condition

When the cut-points were applied to the ASC and combined control groups, sensitivity was 0.95, 95% CI (0.81, 0.99) and specificity was 0.92, 95% CI (0.80–0.97). Specificity was higher when examining just the ASC and non-clinical comparison group [specificity = 1, 95% CI (0.85, 1)], whereas specificity was lower when discriminating ASC from the clinical comparison group [specificity = 0.80, 95% CI (0.57, 0.93)].

#### Relationships of 3Di-Adult DSM-5 Algorithm Scores to Age, IQ and Gender

The A-scale of the 3Di-Adult DSM-5 algorithm was not significantly correlated with age (r_s_ = − .04, p = .704) or IQ (r_s_ = − 0.05, p = .677). The B-scale was also not significantly associated with age (r_s_ = 0.12, p = .291) or IQ (r_s_ = − 0.09, p = .475). We ran two-factor (group by gender) ANOVAs to test for gender effects on A- and B-scale scores. On the A-scale the effect of gender approached, but did not reach significance (p = .065), reflecting a tendency in each group for males to score higher than females. There was no intimation of any gender differences for the B-scale (p = .888). There were no group-by-gender interactions for either the A-scale (p = .566) or the B-scale (p = .691).

## Discussion

This study was a preliminary evaluation of the 3Di-Adult, an informant-based interview schedule specifically designed for assessing ASC in adults based on the DSM-5 criteria. The following findings suggest that the 3Di-Adult is potentially a valuable tool. First, it is reliable within the population investigated here: the scales generated by its diagnostic algorithm are internally consistent and yield scores that have very high inter-rater agreement. Second, the 3Di-Adult has strong content validity, since its questions cover the full range of core autistic features described in DSM-5. Third, criterion validity was demonstrated by the fact that participants with ASC had significantly and substantially higher scores than both non-clinical and clinical comparison participants across all subscales. Furthermore, within the current sample the 3Di-Adult showed high sensitivity (95%) and specificity (92%) when used to identify cases of ASC, even when some comparison participants had non-autistic mental health difficulties. Fourth, the 3Di-Adult appears to possess utility: its use was not impeded by missing data caused by informants struggling to recall relevant information; and it can be administered relatively quickly. On average, interviews for people with ASC took 50 min, whilst comparison interviews tended to be completed within half an hour. Finally, reliability and validity were achieved despite the majority of interviews being conducted over the telephone, suggesting that the 3Di-Adult can be used even when a face-to-face informant interview is not possible.

Despite these encouraging findings, we observe that the 3Di-Adult was not perfect in its classification of participants in the current study. Two of the 39 ASC participants were not picked up by the interview, in both cases because, whilst they scored above threshold on the 3Di-Adult’s A-scale (social communication impairments), they did not have sufficient reported repetitive behaviours to score in the ASC range on the B-scale. This could have arisen because the 3Di-Adult missed their B-scale symptoms, or because they genuinely have social communication difficulties in the absence of significant repetitive behaviours. Such a symptom pattern is found in up to a third of children who met previous (DSM-IV) criteria for ASC (Mandy et al. 2011), and is acknowledged in DSM-5 via its creation of Social (Pragmatic) Communication Disorder (Mandy et al. 2017). Also, four of our 49 comparison participants were incorrectly identified by the interview as having ASC. It is interesting to note that all four were recruited from a specialist ASC assessment clinic where, after careful evaluation, their social and flexibility difficulties had been adjudged not to be attributable to an underlying ASC. This highlights the need for diagnosis to be made by clinician consensus based on multimodal assessment, not just informant report (Pilling et al. 2012).

The current study serves as a preliminary validation of the 3Di-Adult: further work is required to understand fully its psychometric properties and value. Future investigations should prioritise the recruitment of clinical comparison (i.e., non-autistic) participants with mental health difficulties that share phenotypic similarities with ASC, for example, attention deficit/hyperactivity disorder, schizoid personality disorder, schizotypal personality disorder and obsessive compulsive disorder. This would set the 3Di-Adult a more stringent challenge than the one posed by the clinical comparison group in the current study. Also, given that the four false positives we report all came from an ASC assessment service, it will be essential to test comprehensively the 3Di-Adult’s specificity when assessing consecutive referrals to an ASC clinic, which would set the instrument the challenging and ecologically valid task of evaluating complex, marginal cases. Such work will be crucial for conclusively setting optimal diagnostic thresholds for the 3Di-Adult’s DSM-5 diagnostic algorithm.

It is notable that there are no adult informant-report autism assessments that have been tested against consecutive referrals to a specialist autism service. This represents an important gap in the autism literature. Nevertheless, there are two measures currently in use for adult ASC diagnosis, which are wholly based on informant report (Pilling et al. 2012), and which have been evaluated in terms of their ability to discriminate autistic adults from a control group. These are the ADI-R (Lord et al. 1997) and the Asperger Syndrome Diagnostic Interview (ASDI; Gillberg et al. 2001). In addition, the Diagnostic Interview for Social and Communication Disorders (DISCO) has been evaluated for use with adults: this involved testing its sensitivity to ASC in adults, but the absence of an adult control group meant that specificity could not be estimated (Kent et al. 2013). Based on the extant literature, and findings of the current study, the 3Di-Adult has comparable reliability and validity to these measures. The 3Di-Adult is less intensive, and therefore quicker to administer, than the ADI-R and DISCO; and unlike the ADI-R and ASDI, the 3Di-Adult implements current (i.e., DSM-5) criteria. This unique combination of attributes makes the 3Di-Adult a promising tool for use in adult autism assessment services, as part of a multimodal assessment that also includes self-report and direct observation (Pilling et al. 2012). It could be especially useful where the resources are not available routinely to conduct more extensive interviews (such as the ADI-R or DISCO), which take longer and require extensive training to ensure reliable administration. We argue that a gold-standard trial of the 3Di’s ability to identify ASC cases amongst consecutive referrals to specialist ASC assessment services is warranted.

Further, we suggest there is potentially an important role for the 3Di-Adult in general mental health settings. Because Asperger’s syndrome as a diagnostic category was only introduced into DSM-IV and ICD-10 in 1994, anyone born before about 1980 who may have warranted this diagnosis would not have had it available to them during their childhood, and as such have been described as the “lost generation” (Lai and Baron-Cohen 2015). Even since the introduction of Asperger’s syndrome to psychiatric nosology, many children with ASC continue to go unrecognised, and enter adulthood without an appropriate diagnosis (Baron-Cohen et al. 2009). Due to the fact that emotional difficulties regularly co-occur with ASC, many such people present to general mental health services (Mandy and Tchanturia 2015; Davidson et al. 2014). In our clinical experience, the difficulties of adults who present to health services with undiagnosed ASC often go unrecognised, which can limit the effectiveness of the treatment they receive. Our use of a clinical control group (i.e., people without ASC but with mixed mental health difficulties) revealed that scores from the 3Di-Adult are not artificially inflated by the presence of non-autistic psychopathology. Therefore, we propose that the 3Di-Adult will have value in general adult mental health services when autistic difficulties are suspected, to inform clinicians whether a referral for specialist ASC assessment is required.

The 3Di-Adult will likely also be valuable in research where resource-efficient and valid ASC assessment is required, for example when making or confirming ASC diagnosis for treatment trials, case-control studies and epidemiological investigations of the prevalence of ASC in adulthood.

### Limitations and Future Directions

Amongst studies evaluating the psychometric properties of an informant-report autism assessment interview, ours has the largest sample of adults with and without ASC. Nevertheless, the current findings must be judged in the context of the following methodological limitations. First, our clinical comparison group contains participants with a mixture of different clinically diagnosed mental health difficulties such as anxiety, depression, psychosis and personality disorder; and these conditions were not confirmed by an additional structured psychiatric interview schedule. Whilst the nature of this group promotes the ecological validity of our findings by mirroring the nature and diversity of presentations in many clinical mental health settings, it will be valuable to extend our work by investigating 3Di-Adult scores in more homogenous clinical comparison groups whose specific diagnoses have been formally confirmed using standardised measures. In particular it will be interesting to evaluate the 3Di-Adult’s performance and optimal cut points for algorithm scores when discriminating between ASC and psychosis, given the need for valid ASC assessment tools in early-intervention for psychosis services (Davidson et al. 2014). Second, as discussed above, future work will need to be done on consecutive referrals to an ASC assessment service, which would be an ecologically valid and stringent test of the instrument; and should provide information on what cut-points to use in such settings. It is possible that higher cut-off scores that those identified by the current study would be necessary for use within ASC assessment services, given that referral to such a service indicates the presence of some traits or symptoms indicative of ASC.

Third, we only investigated the 3Di-Adult’s psychometric properties in people without an intellectual disability. Given that between a third and half of people on the autism spectrum have an intellectual disability (Loomes et al. 2017), and that it is likely that a substantial number autistic individuals with intellectual disability have reached adulthood without an ASC diagnosis (Shattuck 2006), it will be important to investigate the reliability and validity of the 3Di-Adult in this group. Fourth, researchers were not blind to group which could have biased findings in favour of the 3Di-Adult. Mitigating against this, the second rater for the inter-rater reliability investigation was fully blinded, and registered a very high level of agreement with the original interviewers. Fifth, there were a higher proportion of males in the ASC group than in the control groups. However this is unlikely to have substantially confounded our findings of strong criterion validity, as the group differences we observed were very large, and we found no significant effects of gender on 3Di-Adult scores. Fourth, we did not have sufficient numbers to investigate whether the method of administration (i.e., face-to-face versus telephone) or who acted as the informant (i.e., mother versus other informant) influenced the results. These are important concerns relevant to the efficient design of clinical services and research studies. Future work is required with large samples to compare the 3Di-Adult’s performance for different administration media and informants (Ward-King et al. 2010).

The COSMIN guidelines set out a comprehensive framework for evaluating health measurement instruments, including diagnostic tests (Mokkink et al. 2010). Based on these, we argue that further work is needed to establish the psychometric properties of the 3Di-Adult, in particular its validity. Criterion validity should be further investigated via comparison of 3Di-Adult scores with those from gold-standard measures such as the ADI-R, the Ritvo Autism Asperger’s Diagnostic Scale-Revised (Ritvo et al. 2011) and the Adult Asperger Assessment (Baron-Cohen et al. 2005). Construct validity should also be investigated, which will involve testing whether scores on the 3Di-Adult are consistent with hypotheses based on our current understanding of ASC (Mokkink et al. 2010). In particular structural validity (a subtype of construct validity) should be tested via factor analysis in sufficiently large samples (N > 260), to discover whether the proposed dyadic structure of the 3Di-Adult’s DSM-5 algorithm is empirically supported.

## Conclusions

There is a ‘lost generation’ of adults with ASC who lack a diagnosis because they were not picked up in childhood (Lai and Baron-Cohen 2015). The identification of ASC in adulthood can have a range of positive effects, for example by identifying needs, signposting appropriate treatments, gaining access to services, reducing self-criticism and fostering a positive identity (Shattuck et al. 2012; Punshon et al. 2009; Hurlbutt and Chalmers 2002). Accordingly, it has become a priority for clinicians, researchers, policy-makers and members of the ASC community to improve the recognition of ASC in adults (Department of Health 2015; Howlin and Moss 2012; Pilling et al. 2012). The 3Di-Adult represents a step towards the goal of achieving parity between the standard of child and adult ASC assessment, by providing a reliable, valid and resource-efficient way of collecting diagnostic information from informants. This preliminary validation study indicates promise for clinical use of the 3Di-Adult both as way of informing decision-making in general mental health settings about whether to refer for comprehensive ASC assessment; and indicates the need for further more rigorous study to examine its use as a time-efficient informant report component of a multi-modal ASC assessment in specialist services.

## Footnotes

1 We use the term ‘autism spectrum condition’ (ASC), instead of the DSM-5 term of ‘autism spectrum disorder’ (ASD), as we consider it to be more respectful of neurodiversity. We suggest that the use of ‘ASC’ conveys that people on the autism spectrum show differences that include strengths as well as difficulties. ASC is used here as a direct synonym of ASD, in that it describes those who meet DSM-5 criteria for ASD.
